# Development of the First Shuttle Vector System and Optimization of Transformation in *Selenomonas sputigena*

**DOI:** 10.4014/jmb.2501.01011

**Published:** 2025-04-23

**Authors:** Trinh Thi Nguyen, Yu-Kyung Kim, Duhyun Ko, Jeeyou Kim, Hana Yi, Ye-Ji Bang

**Affiliations:** 1 Department of Microbiology and Immunology, Seoul National University College of Medicine, Seoul 03080, Republic of Korea; 2 Institute of Endemic Diseases, Seoul National University Medical Research Center, Seoul 03080, Republic of Korea; 3 Department of Biomedical Sciences, Seoul National University College of Medicine, Seoul 03080, Republic of Korea; 4 Integrated Biomedical and Life Science, Graduate School, Korea University, Seoul 02841, Republic of Korea; 5 School of Biosystems and Biomedical Sciences, Korea University, Seoul 02841, Republic of Korea

**Keywords:** *Selenomonas sputigena*, shuttle vector, transformation optimization, origin of replication, respiratory microbiome

## Abstract

*Selenomonas sputigena*, an anaerobic bacterium found in the human respiratory tract, has recently gained significant attention due to its dual role in human health — as both a periodontal pathogen and a protective agent against severe asthma. However, the absence of genetic tools for this organism has severely limited our understanding of its molecular mechanisms and therapeutic potential. Here, we report the first successful development of a genetic modification system for *S. sputigena* ATCC 35185. We constructed a shuttle vector carrying the Rep191 replication protein from *Selenomonas ruminantium* and systematically optimized transformation conditions. Through careful optimization of key parameters including DNA quantity, bacterial growth phase, membrane permeabilization, and post-pulse recovery time, we achieved a 22.5-fold improvement in transformation efficiency (from ~2,000 to ~45,000 CFU/μg DNA). The broad applicability of our system was demonstrated through successful transformation of multiple *Selenomonas* species, establishing the first standardized genetic modification system for this genus. We further validated the system's functionality by achieving stable GFP expression in *S. sputigena*, representing the first demonstration of ectopic protein expression in this organism. This work provides essential tools for investigating the molecular basis of *S. sputigena*'s therapeutic effects and pathogenic capabilities, potentially accelerating the development of novel microbiome-based treatments for both respiratory and oral diseases.

## Introduction

*Selenomonas sputigena* has emerged as a bacterium of significant clinical interest due to its complex relationship with human health. Initially isolated from human respiratory tracts [1, 2], this anaerobic Gram-negative bacterium exhibits a remarkable duality in its interactions with the host. While it has been implicated in pathogenic conditions such as periodontal disease and dental caries [4, 5] recent research by Kim *et al*. [3] has revealed its protective role against severe asthma. It has been demonstrated that reduced *S. sputigena* abundance correlates with increased asthma severity in human, and importantly, nasal administration of this bacterium can actively ameliorate asthma symptoms in mouse models. *S. sputigena* treatment significantly reduced IL-5 and IL-13 secreting Th2 cell populations in the mouse lungs, suggesting that this bacterium may protect against asthma by modulating type 2 immune responses that drive allergic inflammation [3]. These findings have opened new avenues of research and suggest *S. sputigena* could potentially be developed as a novel therapeutic agent for asthma treatment. However, the precise molecular interactions and bacterial factors mediating this protective effect remain poorly characterized.

A critical barrier to fully exploiting the therapeutic potential of *S. sputigena* lies in the complete absence of genetic tools for this organism. This fundamental limitation severely hampers our ability to investigate the molecular mechanisms underlying its beneficial effects and pathogenic capabilities. The development of genetic manipulation techniques, particularly transformation methods, is essential for understanding gene functions, regulatory mechanisms, and the intricate dynamics of host-microbe interactions. Such tools would enable the identification and characterization of key bacterial factors involved in asthma protection, potentially leading to new therapeutic strategies. Unfortunately, despite *S. sputigena*’s potential clinical importance, no transformation method currently exists, making genetic manipulation of this organism impossible.

To overcome this fundamental obstacle, we focused on developing a plasmid-based gene expression system. In this study, we report the successful development of a shuttle vector system and establish an electroporation-mediated transformation method for *S. sputigena* ATCC 35185. We systemically optimized transformation efficiency by evaluating key parameters including DNA quantity, cell growth phase, membrane permeabilizing agents, and post-pulse recovery time. Furthermore, we validated our system's applicability in other *Selenomonas* species. The functionality of our system was conclusively demonstrated through the successful visualization of GFP-expressing *S. sputigena*, confirming both the applicability and importance of our system for studying its physiological role within the host. This work represents the first successful development of a vector-host system for *S. sputigena*, providing essential tools that will enable detailed molecular studies of this clinically significant bacterium and potentially accelerate the development of novel microbiome-based therapeutics.

## Materials and Methods

### Materials

Kits for plasmid DNA purification, gel extraction, and PCR clean-up were obtained from Bioneer (Republic of Korea). Yeast extract, tryptone, LB-broth miller, and LB-agar miller were obtained from ForMedium (England). Bacto Tryptic Soy Broth and Bacto Tryptic Soy Agar were obtained from Difco (Becton-Dickinson, USA). Unless otherwise noted, all other chemicals and reagents described in this study were purchased from Sigma-Aldrich (USA).

### Bacterial Strains and Culture Conditions

*Selenomonas sputigena* ATCC 35185 (DSM 20758) was obtained from the DSMZ (German Collection of Microorganisms and Cell Cultures GmbH). The strain was grown in Tryptic Soy Broth supplemented with 5 g/l yeast extract, 1 mg/l menadione (Vitamin K), and 5 mg/l hemin (TSBS). All cultivations were done anaerobically in a vinyl anaerobic chamber (Coy Laboratories, USA) under an atmosphere of 5% CO_2_, 5% H_2_ and 90% N_2_. *Escherichia coli* DH5a was cultured in LB-broth miller and LB-agar miller. Kanamycin was used at concentrations of 50 mg/ml for *E. coli* and 30 mg/ml for all *Selenomonas* species.

### Plasmids and PCR Primers

All plasmids used in this study are listed in Table 1. The shuttle vector pRSR for *S. sputigena* was constructed by cloning the replication protein (rep191) from *S. ruminantium* into a high-copy number plasmid pRSFDuet-1 (Novagen, Australia). All primers and the gene encoding rep191 were synthesized by Bionics (Republic of Korea). Detailed information about strains, and plasmids used in this study is provided in Table 1.

### Antibiotics Sensitivity Test

Antibiotic sensitivity was determined by growing overnight cultures of *S. sputigena* diluted to OD_600_ of 0.1 in TSBS medium supplemented with various antibiotics (kanamycin, streptomycin, chloramphenicol, gentamycin, vancomycin, bacitracin, neomycin, and polymyxin B). These antibiotics were prepared at 64 mg/ml and diluted as needed. Bacterial growth was monitored during anaerobic incubation at 37°C for 72 h.

### Preparation of Electro-Competent Cells and Transformation

Electro-competent cells were prepared following our previously established protocol [10]. Briefly, a single colony was inoculated on TSBS medium with an initial optical density OD_600_ of 0.05-0.1, and grown anaerobically to mid-log phase (OD_600_ of 0.4-0.5) in TSBS medium using a serum bottle or anaerobic chamber. Then, cells were harvested by centrifugation at 5000 rpm for 10 min, and washed with cold electroporation solution (containing 0.25 mM sucrose and 10% glycerol).

The cell pellet was gently pipetted to avoid cell breakage. The washing cells were centrifuged at 5,000 rpm for 10 min and the supernatant was discarded using a pipette. The cell pellets were resuspended in a one-tenth volume of the electroporation solution and the competent cells were ready for electroporation. The electroporation program was used as indicated in the manual of the MicroPulser Electroporator (Bio-Rad, USA). Electrical settings were 2.5 kV, 12,500 V/cm, 5 ms.

### Determination of Transformation Efficiency

The preparation of electro-competent cells and transformation experiments were conducted as described previously [11]. Briefly, the inoculum was transferred into 10 ml prewarmed TSBS and grown up to OD_600_ of 0.4-0.5, corresponding to 1.0-1.2 × 10^7^ colony-forming units (CFUs). Cells were washed twice with ice-chilled buffer (0.25 M sucrose and 10% glycerol) and electroporated with an initial 100 ng of plasmid DNA. Cells were regenerated in 1 ml TSBS medium for 3-4 h (at 37°C, static) and then plated on Tryptic Soy Agar supplemented with 5 g/l Yeast extract, 1 mg/l menadione (Vitamin K), and 5 mg/l hemin (TSAS) with an appropriate antibiotic. Transformation efficiency is determined by the CFU per mg of DNA.

### Visualization of *S. sputigena* Using a Green Fluorescence Protein (GFP)

We selected GFP as the reporter gene for visualization due to several advantages critical for this study: (1) its intrinsic fluorescence eliminates the need for substrate addition, simplifying visualization in anaerobic conditions; (2) its established brightness and stability enable reliable detection even with potentially low-level expression; (3) its compatibility with future fusion protein applications will facilitate protein localization studies; and (4) its proven functionality across diverse bacterial species suggested higher likelihood of successful expression in *S. sputigena*.

To visualize GFP expressed from the pRSR plasmid in *S. sputigena*, the *gfp* gene was cloned into plasmid pRSR under the control of a constitutive promoter derived from *S. sputigena* gene *Selsp_2067*, encoding outer membrane autotransporter barrel domain protein, comprising a 109-bp intergenic region. The GFP-expressing plasmid was transformed into *S. sputigena*, plated on the TSAS agar plate containing kanamycin as the selection marker, and grown anaerobically for 3–4 days. After incubation, the bacterial pellet was carefully collected by scraping from the plate and then resuspended in phosphate-buffered saline (PBS) to ensure a uniform suspension. Next, a small aliquot of the resuspended culture was transferred onto a clean glass microscope slide. A coverslip was then gently placed over the drop to create a thin layer of the sample, which is ideal for imaging purposes. To visualize GFP, a fluorescence microscope (ZEISS Colibri 7, Osunhitech, Republic of Korea) was used with an excitation at 488 nm and an emission at 509 nm. The GFP-expressing bacteria cells were observed at 63X magnification with oil immersion.

### Analytical Methods

Cell density was estimated by measuring an absorbance at 600 nm using a cuvette with a path length of 10 mm and a photometer DEN-600 (Biosan, England), and the viable number of *Selenomonas* on agar plates was determined using CFU method.

## Results

### Construction of Shuttle Vector

**Fig. 1:**
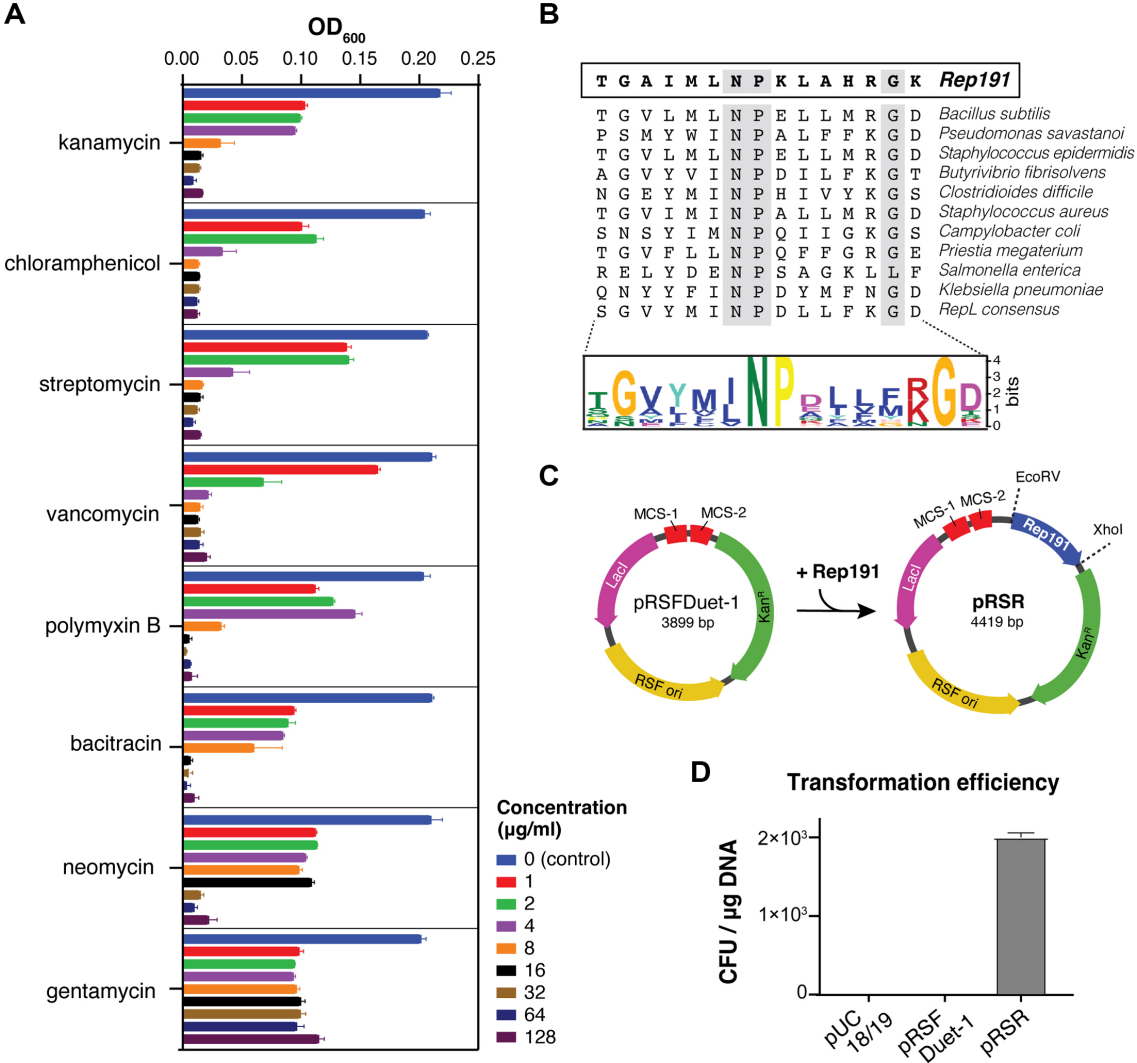
Development of a shuttle vector for *S. sputigena*. (**A**) Antibiotics susceptibility profile of *S. sputigena*. Bacterial growth was measured at OD_600_ after 72 h of anaerobic incubation at 37°C in TSBS medium containing various concentrations of antibiotics (0–128 μg/ml). (**B**) Multiple sequence alignment of 13 plasmid-encoded RepL homologs reveals a conserved motif. The consensus sequence showing the conserved ΦNPX_5_G motif in the helix-turn-helix DNA binding domain. Conserved N, P, and G residues are highlighted in grey. (**C**) Construction of shuttle vector pRSR. The Rep191 gene was inserted into pRSFDuet-1 between *EcoRV* and *XhoI* sites. Plasmid maps show key features including size, replication origins, and antibiotic resistance markers. Kan^R^, kanamycin resistance. (**D**) Transformation efficiency comparison of different plasmids in *S. sputigena* under initial conditions. Only pRSR containing Rep19 yielded transformants, demonstrating the requirement for this replication protein. The experiments were performed in duplicates and the data are shown as means ± SEM.

To develop a system for controlled ectopic gene expression in *S. sputigena*, we first constructed a plasmid capable of replication and maintenance in *S. sputigena* ATCC 35185 (type strain). Initial screening for native plasmids through DNA extraction and agarose gel analysis revealed no cryptic plasmids in this strain (data not shown). We then conducted antibiotic susceptibility testing to identify suitable selection markers, evaluating multiple antibiotics across a concentration range of 1–128 mg/ml. Growth inhibition analysis, measured by OD_600_, demonstrated that kanamycin, chloramphenicol, streptomycin, vancomycin, and polymyxin B exhibited strong inhibitory effects. Bacitracin and neomycin showed moderate inhibition, while gentamycin displayed minimal growth inhibitory activity (Fig. 1A). Based on its robust antimicrobial activity against *S. sputigena* and broad compatibility with other bacterial expression systems, kanamycin was selected as the antibiotic marker for the shuttle vector construction.

Next, we investigated the utility of pUC18/19, a broad-host-range plasmid carrying the ColE1 origin capable of replication in both gram-negative and gram-positive bacteria, although stability may vary among species. Transformation attempts with this plasmid yielded no colonies, indicating that *S. sputigena* requires a specific origin of replication for plasmid maintenance.

A replication protein is essential for plasmid DNA replication [6]. Through analysis of closely related bacteria, we identified several cryptic plasmids in *S. ruminantium* [7-9], including pSRD191, which contains the replication protein known as Rep191. This protein has been classified as a replication protein belonging to the RepL family [9] and thus we aimed to obtain Rep191 and construct a shuttle vector for gene expression in *S. sputigena*. Rep191, consisting of 177 deduced amino acids, shares significant homology with the RepL family of replication proteins found in Firmicutes, including staphylococci and bacilli [9, 11]. We analyzed RepL sequences from diverse plasmid-encoded replication proteins and generated a conserved motif using Multiple EM for Motif Elicitation (MEME) [12] (Fig. 1B). The analysis revealed a strong consensus sequence with a FNPX5G motif in the helix-turn-helix DNA binding domain across all sequences.

The Rep191-encoding DNA sequence was synthesized by Bionics (Republic of Korea), amplified, and cloned into *EcoRV* and *XhoI* sites of the high-copy-number plasmid, pRSFDuet-1, generating pRSR (Fig. 1C). We then evaluated Rep191 functionality in *S. sputigena*. The high-copy number plasmid pRSFDuet-1, carrying RSF 1030 origin and kanamycin resistance gene, served as a negative control. Neither pUC18/19 nor pRSFDuet-1 produced transformant colonies (Fig. 1D), confirming the requirement for a specific replication origin protein. In contrast, the Rep191-containing pRSR plasmid successfully generated colonies on kanamycin-supplemented TSAS agar.

### Optimization of Transformation Conditions for *Selenomonas sputigena* ATCC 35185

After shuttle vector construction, we sought to optimize transformation conditions for *S. sputigena*, as transformation protocols for the *Selenomonas* genus are poorly characterized. Key parameters requiring optimization included washing solutions, pulse settings, and cell growth conditions. Given that cell walls of *Selenomonas* are approximately twice as thick as those of *E. coli* [2] we hypothesized that electroporation would be more effective than chemical transformation using heat. Indeed, chemical transformation yielded no colonies for any tested plasmid (data not shown), leading us to focus on electroporation optimization. When tested with pulse strengths ranging from 1.8kV to 3.0kV, only the 2.5kV pulse resulted in colony formation. Similarly, among the recovery media tested (SOB, SOC, LB, and TSBS), colonies appeared only when cells were recovered on TSBS after electroporation at 2.5 kV. This suggested that *S. sputigena* requires a specific recovery medium, with TSBS being necessary for successful transformation. Next, we systematically optimized four parameters: (i) plasmid DNA amount, (ii) bacterial growth phase, (iii) cell membrane permeabilizing agents, and (iv) recovery time after electroporation.

### Effect of Plasmid DNA Amount and Bacterial Growth Stage on Transformation Efficiency

**Fig. 2:**
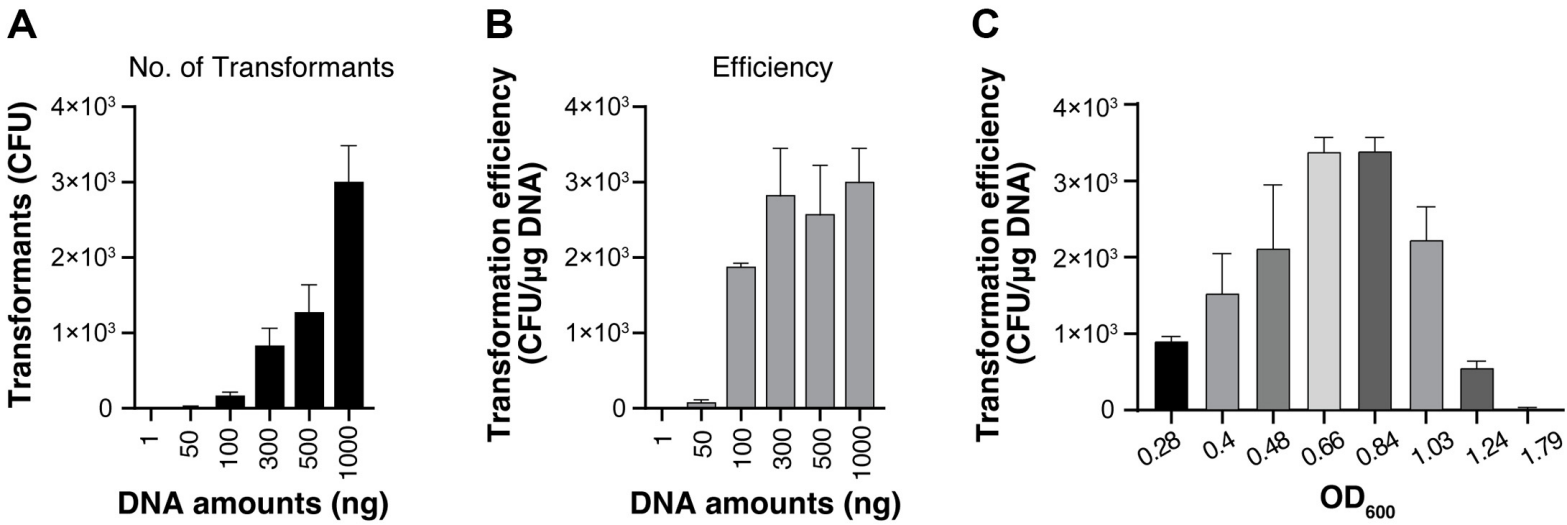
Optimization of DNA amount and growth phase for *S. sputigena* transformation. (**A**) Effect of plasmid DNA quantity on the transformation yield in *S. sputigena*. Total number of transformants obtained using varying amounts of plasmid DNA (1–1,000 ng). (**B**) Transformation efficiency calculated as CFU/μg DNA for different DNA amounts, showing optimal efficiency at 300 ng plasmid DNA. (**C**) Impact of bacterial growth phage on transformation efficiency. *S. sputigena* cells grown in TSBS were harvested at different optical densities (OD_600_ 0.3–1.8) and transformed with 300 ng plasmid DNA. All experiments were performed in duplicates and data represent means ± SEM.

We tested various amounts of plasmid DNA, ranging from 1 ng to 1,000 ng, to determine the optimal quantity for electroporation and to calculate the transformation efficiency. The number of transformants increased proportionally with DNA amount, reaching approximately 3,000 CFU at 1,000 ng (1 mg) of DNA (Fig. 2A). However, transformation efficiency (CFU/mg DNA) showed a different pattern, reaching a plateau between 300–1,000 ng of input DNA, with all values in this range achieving approximately 2,500–3,000 CFU/mg DNA (Fig. 2B). Since 300 ng of DNA provided comparable efficiency to higher amounts while conserving plasmid DNA, we selected this quantity for subsequent experiments.

Growth phase effects were evaluated from early exponential to stationary phase using 10 ml TSBS medium cultures at OD_600_ values between 0.3 and 1.8. Notably, mid-log phase cells (OD_600_ 0.6–0.8) showed significantly enhanced transformation frequency, with transformation efficiency approximately two-fold higher than early log phase (OD_600_ 0.3) and 80-fold greater than late stationary phase (OD_600_ 1.79) (Fig. 2C). These optimal conditions (OD_600_ 0.6–0.8 and 300 ng DNA) were applied in subsequent optimization steps.

### Effect of cell Membrane Permeabilizing Agent

**Fig. 3:**
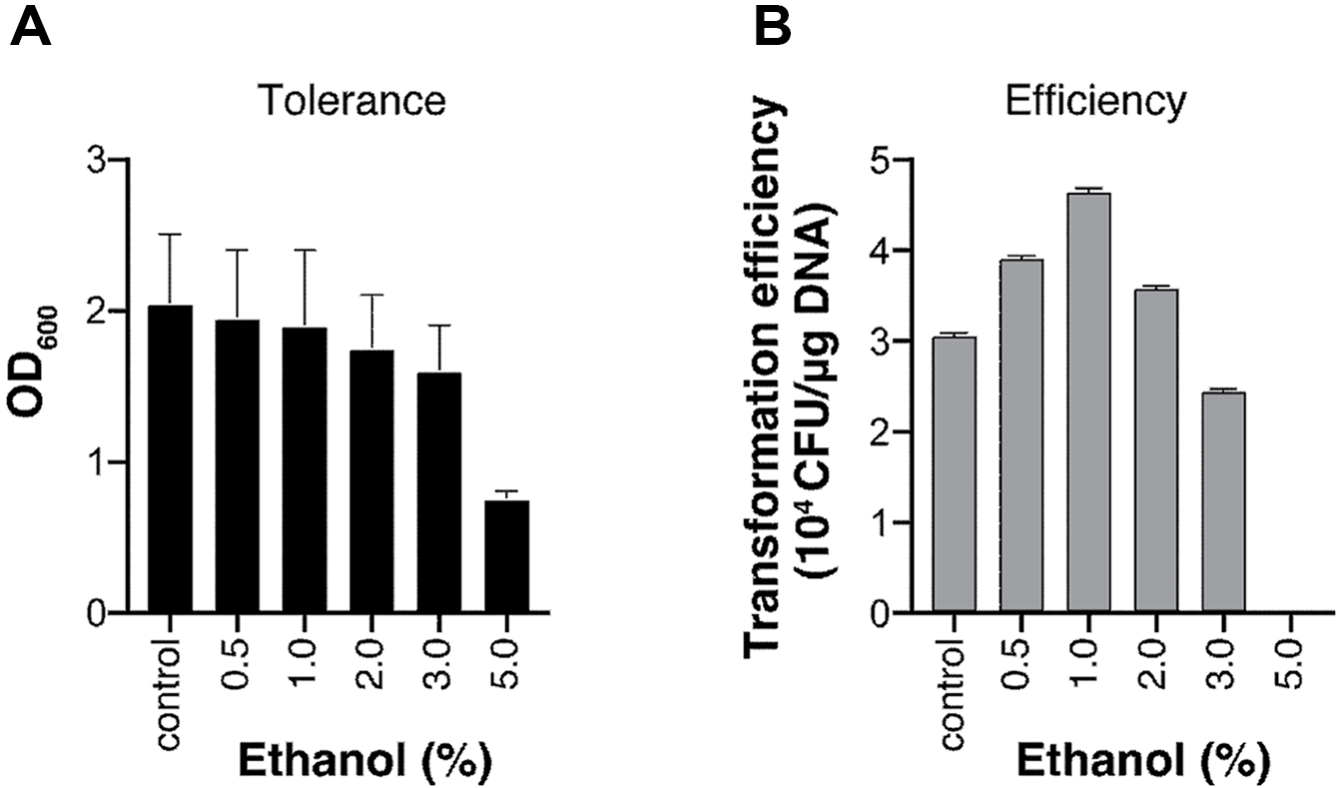
Effect of ethanol pretreatment on transformation efficiency. (**A**) Ethanol tolerance of *S. sputigena*. Growth was monitored at OD_600_ in the presence of various ethanol concentrations (0.5–5% v/v). (**B**) Impact of ethanol pretreatment on transformation efficiency. Competent cells were treated with different ethanol concentrations for 10 min. before electroporation with 300 ng pRSR. The results were shown as the number of transformants/μg plasmid DNA. All experiments were performed in duplicates and the data are shown as means ± SEM.

Ethanol acts as a membrane-solubilizing agent that improves the electroporation efficiency in certain bacteria by modifying the characteristics of their cell membranes [14-17]. The mechanism involves ethanol binding to the lipid-water interface of phospholipid bilayers, weakening the membrane’s hydrophobic barrier and increasing its fluidity and pore size [18-21]. However, different bacteria exhibit varying levels of tolerance to ethanol’s toxicity [22]. We thus first assessed the ethanol tolerance of *S. sputigena* by testing its growth in the presence of various ethanol concentrations. Growth analysis revealed a gradual decrease in OD_600_ with increasing ethanol concentration, with significant inhibition observed at 5.0% ethanol (Fig. 3A).

We then evaluated the effect of ethanol pretreatment on transformation efficiency. Various concentrations of ethanol were introduced to the competent cell-DNA mixture ten minutes before electroporation. The transformation efficiency showed a clear concentration-dependent pattern. Treatment with 1.0% ethanol markedly enhanced transformation efficiency, showing approximately 1.5-fold increase compared to the control. However, the efficiency progressively declined at higher concentrations, dropping below control levels at 3.0% ethanol, likely due to the cumulative toxic effects of ethanol on membrane integrity (Fig. 3B).

### Effect of Recovery Time on Transformation

**Fig. 4:**
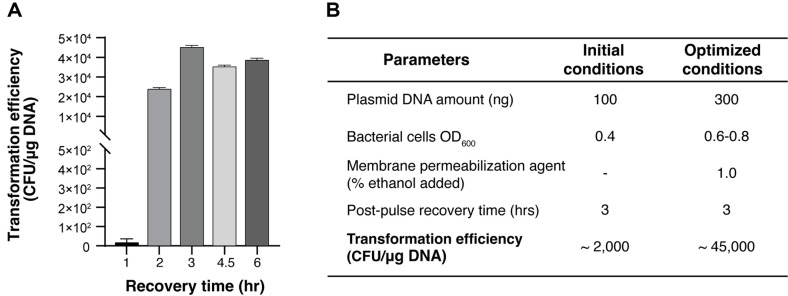
Recovery time optimization and summary of optimized conditions. (**A**) Effect of post-electroporation recovery time on transformation efficiency. Cells were allowed to recover for varying periods (1–6 h) before plating. Experiments were conducted twice independently and the data are shown as means ± SEM. (**B**) Comprehensive comparison of transformation parameters and their optimization. The table shows key parameters and their values under initial versus optimized conditions, demonstrating a 22.5-fold increase in transformation efficiency.

Post-pulse recovery is a crucial step for allowing competent cells to recover from electric shock and express the antibiotic resistance genes present on the plasmid. We investigated the optimal recovery period for electroporated of *S. sputigena* by varying incubation times from 1 to 6 h. The transformation efficiency showed a clear time-dependent pattern, with minimal efficiency at 1 h followed by a sharp increase, reaching maximum efficiency at 3 h. Longer recovery periods of 4.5 and 6 h maintained high but slightly decreased efficiency (Fig. 4A). This suggests that a 3-h recovery period is optimal for *S. sputigena* transformation.

Through systematic optimization of multiple parameters, including DNA amount (300 ng), bacterial growth phase (OD_600_ 0.6-0.8), ethanol pretreatment (1.0%), and recovery time (3 h), we achieved a dramatic improvement in transformation efficiency from initial conditions (~2,000 CFU/mg DNA) to optimized conditions (~45,000 CFU/mg DNA), representing a 22.5-fold increase (Fig. 4B). This optimized protocol provides a robust foundation for genetic manipulation of *S. sputigena*.

### Validation of Vector System across Diverse *Selenomonas* Species

**Fig. 5:**
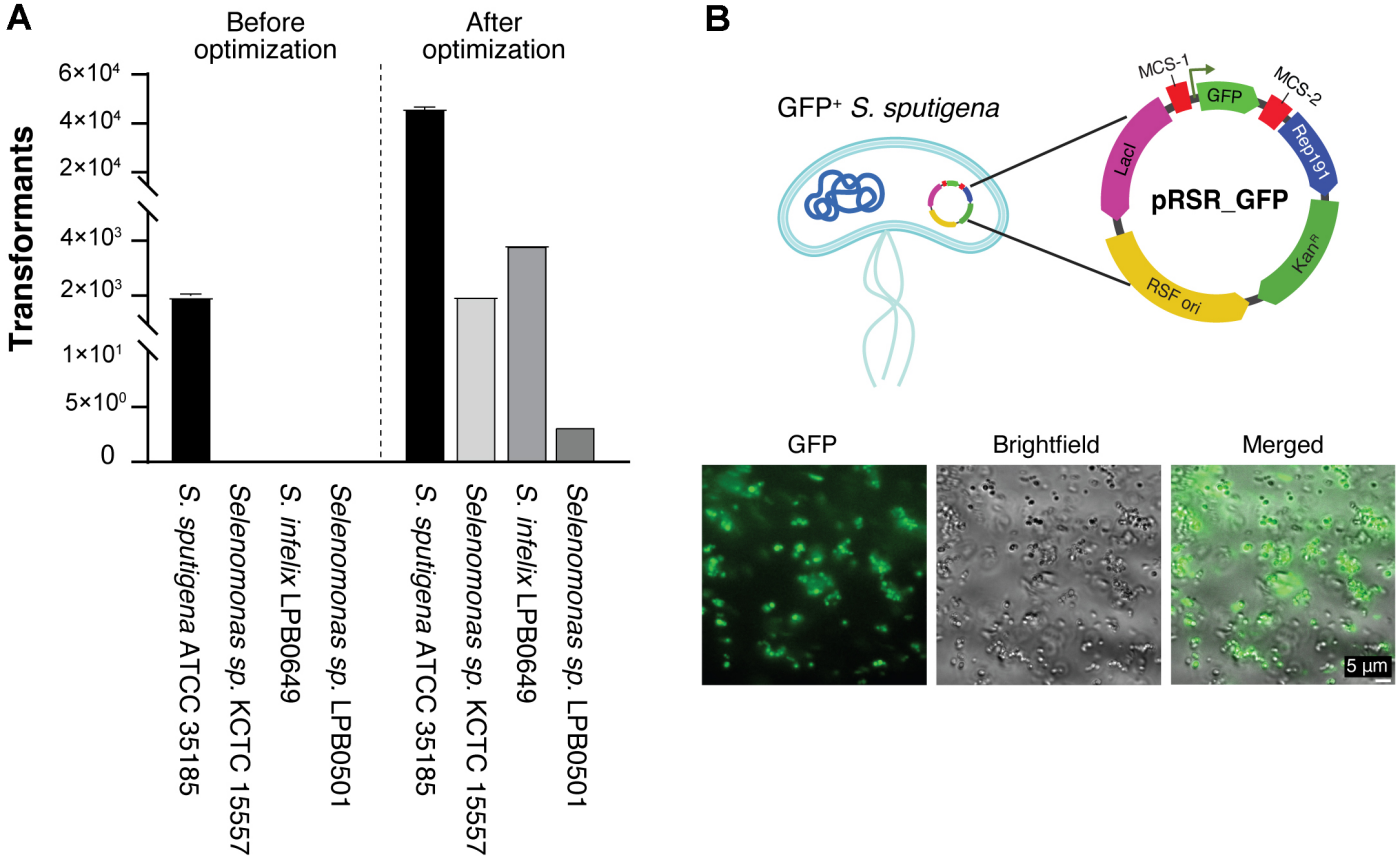
Broad applicability and functional validation of the optimized genetic manipulation system. (**A**) Cross-species application of the shuttle vector system. Transformation efficiency was evaluated in multiple *Selenomonas* species (*S. sputigena* ATCC 35185, *Selenomonas* sp. KCTC 15557, *S. infelix* LPB0649, and *Selenomonas* sp. LPB0501) under both initial and optimized conditions. The optimized protocol significantly improved transformation efficiency across different species. (**B**) Functional validation of the shuttle vector system through ectopic protein expression. *Top*: Plasmid map of pRSR_GFP showing the key features including GFP gene insertion, Rep191, and antibiotic resistance marker. Bottom: Fluorescence microscopy images demonstrating successful GFP expression and visualization of GFP-expressing *S. sputigena*.

To evaluate the broader applicability of our vector system and transformation protocol, we tested transformation efficiency across multiple *Selenomonas* species available in laboratory, including *Selenomonas* sp. KCTC 15557, *S. infelix* LPB0649, and *Selenomonas* sp. LPB0501. Using our optimized procedure for *S. sputigena*, we successfully obtained transformants in all tested species, although with varying efficiencies (Fig. 5A). While *Selenomonas* sp. LPB0501 showed relatively lower transformation efficiency, the successful transformation across multiple species demonstrates the versatility of our vector system.

This broad compatibility is particularly significant as it establishes the first standardized genetic manipulation system applicable across the *Selenomonas* genus. The successful transformation of different *Selenomonas* species suggests that the Rep191-based replication system and our optimized protocol could potentially be adapted for other members of the *Selenomonadaceae* family, opening new possibilities for genetic studies in these historically challenging organisms.

### Visualization of Green Fluorescence in *S. sputigena* Cells

To demonstrate the practical utility of our vector system for gene expression studies, we generated a recombinant *S. sputigena* strain expressing GFP as a reporter gene. Fluorescence microscopy analysis revealed robust GFP expression under the control of a constitutive promoter in pRSR_GFP (Fig. 5B). The successful expression of GFP is particularly significant as it represents the first demonstration of heterologous protein expression in *S. sputigena*.

This achievement validates not only the functionality of our shuttle vector system but also confirms that the basic transcriptional and translational machinery of *S. sputigena* can effectively process foreign genes. The stable expression of GFP establishes our system as a reliable platform for future genetic manipulation studies, including but not limited to gene regulation analysis, protein localization studies, and investigation of host-microbe interactions.

## Discussion

The development of genetic tools for clinically relevant bacteria represents a critical step in understanding their role in human health and disease. While electroporation typically achieves transformation efficiency of up to 1×10^7^ CFU/mg DNA in model organisms like *E. coli* [26-28], the genetic manipulation of *Selenomonas* species has remained a significant challenge due to their strict anaerobic nature and distinctive cell wall characteristics, which is nearly twice the thickness of *E. coli* [2].

Our study presents the first successful development of a genetic manipulation system for *S. sputigena*, addressing a crucial gap in the field. Through systematic optimization of multiple parameters, we achieved a 22.5-fold improvement in transformation efficiency (from 2,000 to 4.5 × 10^4^ CFU/mg DNA) (Fig. 4B). While this efficiency may not match that of established model organisms, it represents a significant breakthrough for *Selenomonas* research, comparable to optimization achievements in other challenging bacterial species. For instance, transformation efficiency improvements ranging from 100-fold in *Bifidobacterium bifidum* (reaching 10^5^ CFU/mg DNA) [17] to 1,000-fold in *Bacillus amyloliquefaciens*, (reaching 8.9 × 10^5^ CFU/mg DNA) [29] have been reported. Similarly, *Bacteroides fragilis* showed 2 to 900-fold improvements through plasmid optimization, ultimately achieving 10^5^ CFU/mg DNA [11].

The broad applicability of our shuttle vector system across multiple *Selenomonas* species (Fig. 5A) marks a particularly significant advancement, as it establishes the first standardized genetic manipulation platform for this genus. This breakthrough is especially timely given the emerging recognition of *S. sputigena*'s dual role in human health - from its involvement in periodontal disease to its newly discovered protective effects against severe asthma [3-5]. Our genetic manipulation system opens several critical research avenues. Future research will focus on identifying factors associated with both the beneficial and potentially pathogenic roles of *S. sputigena* by studying its dynamic interactions with host cells. Our vector system provides the foundation for future genetic manipulation strategies, including gene expression studies and potentially gene inactivation approaches. It provides tools to explore the bacterium's unique metabolic capabilities, particularly those potentially involved in modulating host immune responses. Our system also facilitates investigation of colonization mechanisms and niche adaptation factors that may explain the context-dependent effects of *S. sputigena* in different body sites and disease states, such as in asthma and periodontal disease. By enabling protein expression and visualization, as demonstrated by our GFP expression system (Fig. 5B), our system will allow studies of bacterial-host cell interactions. Exploring these complex interactions may reveal crucial mechanisms underlying the protective and potentially pathogenic effects of *S. sputigena*, advancing our understanding of how this organism can play different roles in human disease depending on the microenvironment.

While the current transformation efficiency may be insufficient for certain advanced genetic manipulations, such as genome integration generally requiring efficiencies of 1.0 × 10^-4^ to 1.0 × 10^-6^ per plasmid with homologous regions under 1 kb [13] our work establishes a crucial foundation for further optimization. Future improvements could explore additional parameters such as cell wall weakening agents (NaCl, Glycine) and the R-M system [30-34]. More importantly, our current system already enables a wide range of molecular studies that were previously impossible, including the investigation of virulence factors, metabolic pathways, colonization, and therapeutic mechanisms.

This work represents a significant technological advancement that bridges a critical methodological gap in *Selenomonas* research. The ability to genetically manipulate *S. sputigena* will be particularly valuable for investigating its protective mechanisms against asthma, potentially accelerating the development of novel microbiome-based therapeutics. Furthermore, our system provides essential tools for studying other clinically relevant aspects of *Selenomonas* biology, from its role in oral health to its broader implications in human disease. As the importance of the human microbiome in health and disease becomes increasingly apparent, these genetic tools will be instrumental in unraveling the complex interactions between *Selenomonas* species and their hosts, potentially leading to new therapeutic strategies for both respiratory and oral diseases.

## Figures and Tables

**Table 1:** Bacterial strains and plasmids are used in this study.

Resource	Source	Description
Bacterial strains		
*E. coli* DH5α	DSMZ, Germany	Cloning host
*S. sputigena* ATCC 35185	DSMZ, Germany	Wild type strain
*Selenomonas* sp. KCTC 15557 (ATCC33150)	KCTC, Korea	Wild type strain
*S. infelix* LPB0649	Korea University, Korea	Wild-type, lab-isolated strain
*Selenomonas* sp. LPB0501	Korea University, Korea	Wild-type, lab-isolated strain
Plasmids		
pUC18/19	[35]	ColE1-*ori*; pRO1614-*ori*; broad-host-range cloning vector; Amp^R^
pRSFDuet-1	Novagen, Australia	pRSF1030 replicon (also known as NTP1), *lacI* gene, Kan^R^
pRSR	This study	Replication gene rep191 cloned into pRSFDuet-1, Kan^R^
pRSR_GFP	This study	pRSR, expressing GFP under the control of a constitutive promoter
